# Supplementary material for: Antimicrobial activity of iron-depriving pyoverdines against human opportunistic pathogens
Source: eLife. 2024 Dec 18;13:RP92493. doi: 10.7554/eLife.92493 (PMC11655065; doi:10.7554/eLife.92493)

**Supplementary File 1**

Antimicrobial activity of iron-depriving pyoverdines against human opportunistic pathogens

Vera Vollenweider^1^, Karoline Rehm^2^, Clara Chepkirui^3^, Manuela Pérez-Berlanga^1^, Magdalini Polymenidou^1^, Jörn Piel^3^, Laurent Bigler^2^, Rolf Kümmerli^1^

^1^Department of Quantitative Biomedicine, University of Zurich, Zurich, Switzerland

^2^Department of Chemistry, University of Zurich, Zurich, Switzerland

^3^Institute of Microbiology, Eidgenössische Technische Hochschule (ETH) Zurich, Zurich, Switzerland

**Supplementary File 1a**

**Supplementary File 1b**

**Supplementary File 1c**

**Supplementary File 1d**

**Supplementary File 1e**


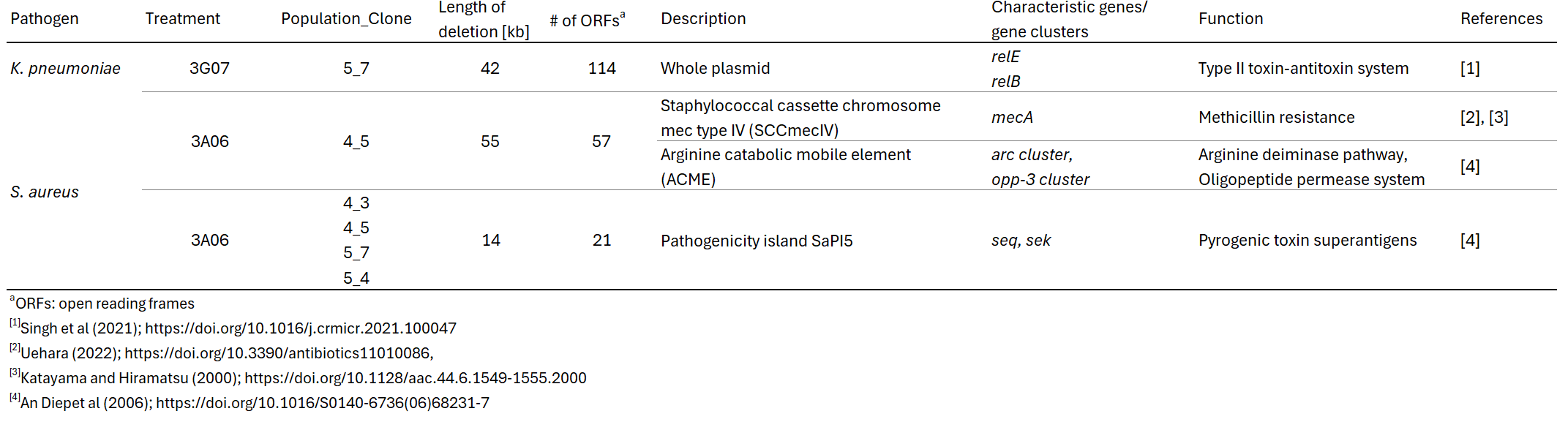

Supplement: Supplementary file 1. [file elife-92493-supp1.docx]
